# Evaluating the pharmacological response in fluorescence microscopy images: The Δ*m* algorithm

**DOI:** 10.1371/journal.pone.0211330

**Published:** 2019-02-13

**Authors:** Ana I. Gómez, Marcos Cruz, Juan F. López-Giménez

**Affiliations:** 1 Department of Mathematics, Statistics and Computer Science, Universidad de Cantabria, Santander, Spain; 2 Institute of Parasitology and Biomedicine “López-Neyra”, CSIC, Granada, Spain; University of Campinas, BRAZIL

## Abstract

Current drug discovery procedures require fast and effective quantification of the pharmacological response evoked in living cells by agonist compounds. In the case of G-protein coupled receptors (GPCRs), the efficacy of a particular drug to initiate the endocytosis process is related to the formation of endocytic vesicles or endosomes and their subsequent internalisation within intracellular compartments that can be observed with high spatial and temporal resolution by fluorescence microscopy techniques. Recently, an algorithm has been proposed to evaluate the pharmacological response by estimating the number of endosomes per cell on time series of images. However, the algorithm was limited by the dependence on some manually set parameters and in some cases the quality of the image does not allow a reliable detection of the endosomes. Here we propose a simple, fast and automated image analysis method—the Δ*m* algorithm- to quantify a pharmacological response with data obtained from fluorescence microscopy experiments. This algorithm does not require individual object detection and computes the relative increment of the third order moment in fluorescence microscopy images after filtering with the Laplacian of Gaussian function. It was tested on simulations demonstrating its ability to discriminate different experimental situations according to the number and the fluorescence signal intensity of the simulated endosomes. Finally and in order to validate this methodology with real data, the algorithm was applied to several time-course experiments based on the endocytosis of the mu opioid receptor (MOP) initiated by different agonist compounds. Each drug displayed a different Δ*m* sigmoid time-response curve and statistically significant differences were observed among drugs in terms of efficacy and kinetic parameters.

## Introduction

Recent advances in microscopy technologies have made possible to acquire large numbers of images that require new data analysis methodologies to gain insight on complex biological processes. In this sense, automatic image analysis methods aim to provide quantitative measurements from acquired images with minimal human supervision. They are of greatest interest either for drug discovery processes to quantify biochemical and/or cellular effects produced by a given compound [1] as in other applications such as diagnosis, morphology studies or gene function [2].

Here we focus on drugs inducing the formation of endosomes, which are internalizing vesicles from the cell membrane to the cytoplasm. This process can be observed in fluorescence microscopy images of living cells as a result of G-protein coupled receptors (GPCR) activation by agonist compounds. In pharmacology the capacity of an agonist to promote a response through a given receptor in a specific tissue is known as efficacy. Therefore, quantifying this response might be useful to evaluate and compare the pharmacological properties of different drugs, i.e. affinity to bind to a specific site and/or potency and efficacy to evoke a biological response. Nevertheless, there is a lack of quantitative methods to accurately evaluate the agonist efficacy to promote endocytosis based on fluorescence microscopy imaging. ArrayScan technology [3, 4] was formerly proposed to quantitatively evaluate GPCR endocytosis by analysing the appearance and intensity of fluorescent receptor aggregates inside the cell. However, it was based on “Top Hat” filter which do not give truly satisfactory results with biological images [5]. Other reports were proposed to analyse the time course of the process [6] but at the expense of using sensitive imaging technology that required highly complex acquisition conditions.

Current fluorescence microscopy technologies permit to observe this cellular process with high spatial and temporal resolution. This pharmacological response can be characterised by different parameters related to the generation of endosomes including their number, the intensity of the associated fluorescence signal or the distribution of their sizes as suitable options to evaluate the pharmacological properties of a drug.

Several methods have been proposed for spot detection in fluorescence microscopy images [7–10]. In the latter work, the Q-endosomes algorithm was proposed to quantify the number of endosomes generated upon activation of the mu opioid (MOP) receptor in images from living cells obtained by epifluorescence microscopy [10]. The algorithm consisted on several steps including Gaussian filtering, local maxima identification above a given local background threshold, *ν* = 90%, and correlation of the selected maxima with a 2D-Gaussian function of a given standard deviation, *σ* = 2.30. Finally, the local maxima with correlation above a given threshold, *ρ* = 0.75, were counted as endosomes. The obtained experimental data resulted in some significant differences in terms of number of endosomes per cell depending on the drug used to initiate receptor endocytosis. However, this algorithm presents room for improvement as we have observed ill-conditioned behaviour with respect to the three manually set parameters, i.e. *ν*, *σ* and *ρ*. Furthermore, the algorithm is not fully automated as it requires manual or independent cell counting that may be biased. Finally, only the number of endosomes and not their brightness is quantified. The Q-endosomes algorithm assumes that the endosomes have a Gaussian-like shape in the images and that their average size is constant over time.

Here we propose a new algorithm to quantify pharmacological responses based on receptor endocytosis taking into account both, the number of endosomes and their brightness. The algorithm, hereafter the Δ*m* algorithm, might be applied to a set of time-course images. It provides a global dimensionless quantification for the entire image which can be used to compare among different experiments. Moreover it allows to detect and discard experiments with systematic artifacts. It is fast and relies only on a single parameter namely the mean endosome size in pixels. This parameter has to be set manually and is assumed to be constant over time as it was in the Q-Endosomes algorithm. However the results do not strongly depend on this assumption nor on small variations of the size parameter. The new algorithm is presented and justified in section Outline of the Δ*m* Algorithm. It is tested on simulated images in section Algorithm evaluation with simulated endosomes, and in section Application to real experiments on real images. The discussion is presented in section Discussion.

## The Δ m algorithm

### Definitions and notation

We define the following notation

- $\vec{x}=\left(x,y\right)$: Position vector on the image plane. The *z*-axis is perpendicular to the image plane.
- *t*: Time at which the image is taken in minutes. The agonist compound is added at time *t* = 0.
- $I_{t}\left(\vec{x}\right)$: a set of *n*_*t*_ time-course images, abbreviated as $\left\{I_{t}\}=\{I_{0},I_{1},\ldots ,I_{n_{t}-1}\right\}$.
- *B*_*t*_: Region of interest for image *I*_*t*_.
- *F*_*i*_(*x*, *y*): Fluorescence signal at position (*x*, *y*) from the *i*th endosome of size *γ*_*i*_ centered at (*x*_*i*_, *y*_*i*_). We assume that it can be modeled as a 2D-Gaussian function:
  $$\begin{array}{c} F_{i}\left(x,y\right)=A\;\text{exp}(-\frac{{\left(x-x_{i}\right)}^{2}+{\left(y-y_{i}\right)}^{2}}{2\gamma_{i}^{2}}). \end{array}$$ (1)
- *γ*: Average endosome size parameter, *γ* > 0. In our images *γ* ≈ 2 pixels.
- *A*: Amplitude (or intensity parameter) *A* > 0,
- *s*: Standard deviation computed for *I*_0_ in region *B*_0_.
- $I_{t}^{\prime }\left(\vec{x}\right)$: Image $I_{t}\left(\vec{x}\right)$ after convolving with the LoG filter. To lighten the notation we use the abbreviation $I_{t}^{\prime }$.
- *R*: scale parameter *R* > 0 of the Laplacian of Gaussian (LoG) filter.
- *A*′: Amplitude of a filtered endosome
- *s*′: Standard deviation computed for $I_{0}^{\prime }$ in region *B*_0_.
- λ: Amplification,
  $$\begin{array}{c} \lambda =\frac{A^{\prime }/s^{\prime }}{A/s}. \end{array}$$ (2)
- *R*_*opt*_: LoG-filter scale maximizing the amplification, λ in the region of interest *B*_0_.
- *m*_*t*_: Third order moment in region *B*_*t*_ of image $I_{t}^{\prime }$.

### Outline of the Δ*m* algorithm

**Fig 1:**
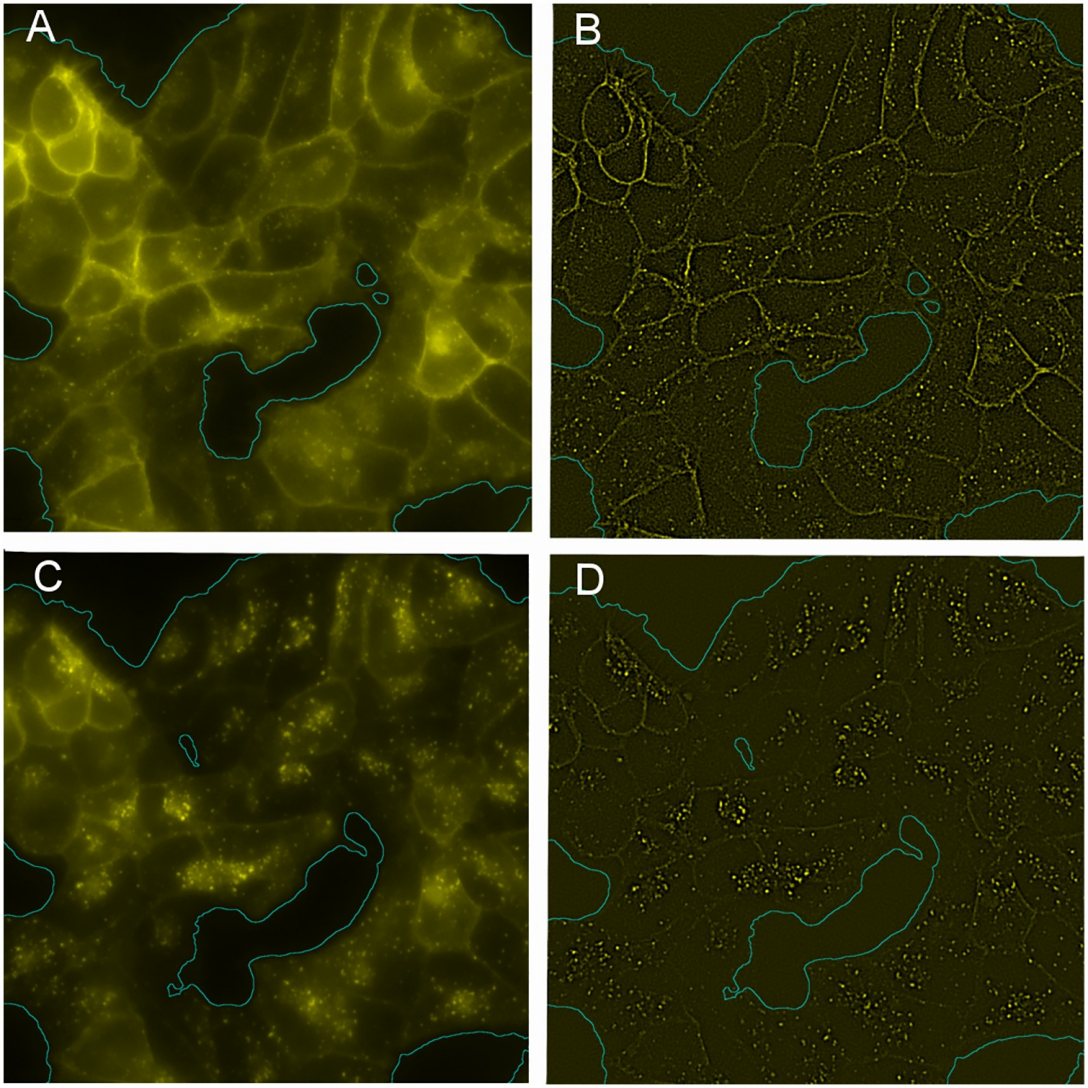
Fluorescence microscopy images. Fluorescence microscopy images corresponding to cells treated with DAMGO (10*μM*) 4 (A) and 12 minutes (C) before picture acquisition. Region of interest segmentation was performed using a simple thresholding method combined with a gaussian blur filter (See Segmentation of the Region of Interest). (B) and (D) show the same images as in (A) and (C) respectively, after convolution with a LoG filter of scale *R* = 1.75 pixels.

The basic steps of this algorithm are:

1. Find the region of interest, *B*_*t*_, for each image of the set {*I*_*t*_} (see Fig 1A and 1C). Empty regions with no cells are excluded as explained in section Segmentation of the Region of Interest.
2. Choose or estimate the average endosome size *γ*.
3. Find the optimal LoG scale *R*_*opt*_ for a given average endosome size *γ*. The scale is selected maximizing the amplification, λ, in the region of interest *B*_0_ (see section Scale parameter selection: Endosome amplification).
4. Convolve the images {*I*_*t*_} with the LoG filter at scale *R*_*opt*_, obtaining a set of filtered images $\left\{I_{t}^{\prime }\right\}$ (see Fig 1B and 1D).
5. Calculate the third order moment, *m*_*t*_, of the *n*_*pix*_ pixels in region *B*_*t*_ of each image $I_{t}^{\prime }$:
  $$\begin{array}{cc} m_{t}=\frac{1}{n_{pix}}\sum_{\vec{x}\in B_{t}}(I_{t}^{\prime }\left(\vec{x}\right)-\bar{I_{t}^{\prime }}{)}^{3},\; & \bar{I_{t}^{\prime }}=\frac{1}{n_{pix}}\sum_{\vec{x}\in B_{t}}I_{t}^{\prime }\left(\vec{x}\right), \end{array}$$ (3)
  $$\begin{array}{c} \Delta m_{t}=\frac{m_{t}-m_{0}}{m_{0}}. \end{array}$$ (4)

A set of {Δ*m*_*t*_} values is obtained for each experiment which is used to quantify the pharmacological response.

### Justification of the algorithm

The Q-endosomes algorithm [10] filtered the images with a 2D-Gaussian in order to reduce instrumental noise. The Δ*m* algorithm instead uses the LoG filter, since [11] showed that the LoG filter is the optimal pseudo-filter used to detect 2D-Gaussian shaped objects for a wide range background images. It is therefore widely used in spot detection [9, 12]. It amplifies spots with central symmetry reducing the background noise if the scale is appropriately chosen. In addition, constants and gradients are cancelled out after convolution with the LoG filter since it is compensated, i.e. the integral below the curve is zero.

The main difference between images *I*_*t*_ and *I*_0_ is the fluorescence signal produced by the endosomes that are present at time *t* but not at *t* = 0. This fluorescence signal generates an increment in the asymmetry of the histogram of *I*_*t*_ with respect to *I*_0_ which can be measured comparing the second, third and fourth order moments of images *I*_*t*_ and *I*_0_. Hence, the relative increment of these three moments are potential candidates to quantify the pharmacological response. Checking their performance with the simulated images of section Algorithm evaluation with simulated endosomes the third order moment showed slightly better results than the fourth order moment and considerably better results than the second order moment.

The Δ*m* algorithm quantifies the pharmacological response of the living cells in each image *I*_*t*_ through one single value, Δ*m*_*t*_. The regions without cells (see for instance Fig 1) can affect the third order moment calculation, blurring the desired quantification. Therefore, Δ*m*_*t*_ is computed considering only pixels in region of interest *B*_*t*_.

The algorithm only depends on a single parameter, namely average endosome size, *γ*, which we set to *γ* = 2 pixels in the considered experiments. This was the value given in the Supplemental Data of [10] based on manual measurements of 150 endosomes. We checked that small variations in *γ* do not significantly affect our results (see Algorithm evaluation with simulated endosomes).

### Segmentation of the region of interest

A segmentation method is needed to identify the region of interest *B*_*t*_. A global, histogram-derived thresholding method was used in which the threshold was automatically calculated using the minimum algorithm [13, 14]. This simple method was sufficient to segment the images considered here. However, in other cases some advanced techniques might be necessary (see [15], and [16] for a detailed discussion).

The images considered here (see section Application to real experiments) were previously blurred with a gaussian filter of radius equal to 3 pixels, that performed well in most of the image sets. In cases where no solution was obtained for the threshold, the blur radius was iteratively increased by one pixel until a maximum of 10 pixels. The region of interest *B*_*t*_ was defined by the pixels above the obtained threshold, and additionally it was required to contain at least 50% of the pixels of the image, otherwise it was rejected. In the few cases where no segmentation was found, *B*_*t*_ was set equal to the entire image.

For each set {*I*_*t*_}, the corresponding minimum area region of interest, *B*_*min*_ of the set {*B*_*t*_} was selected. To avoid noticeable variations due to segmentation inconsistencies, *B*_*t*_ which differ from *B*_*min*_ in more than 5% of the total pixels of the image were set to *B*_*min*_.

### Scale parameter selection: Endosome amplification

The LoG filter of scale parameter *R*, can be expressed as follows (Eq 1 of [12]):

$$\begin{array}{c} \psi \left(x,y\right)=\frac{1}{2\pi R^{4}}\;(2-\frac{x^{2}+y^{2}}{R^{2}})\text{exp}[-\frac{\left(x^{2}+y^{2}\right)}{2R^{2}}]. \end{array}$$ (5)

In this work, we use a Fiji plugin with a kernel adapted from [17] by increasing the size to int(4*R*) × 2 + 1 pixel.

The Δ*m* algorithm selects the scale parameter *R* maximizing amplification λ defined by Eq 2. Consider a background image *I*_0_ of a time-course experiment {*I*_*t*_}. The calculation of standard deviations, *s* and *s*′ are straightforward. The amplitude ratio *A*′/*A* can be calculated adapting Eq 9 from [18]:

$$\begin{array}{c} \frac{A^{\prime }}{A}=\frac{2\gamma^{2}}{{\left(\gamma^{2}+R^{2}\right)}^{2}}. \end{array}$$ (6)

Note that the LoG definition used here, and the one in Eq 5 of [18] with *n* = 1, differ by a factor of *R*^2^/2.

*R*_*opt*_ can be estimated by calculating λ for a set of scales {*R*}, selecting the one that yields a maximum λ.

For the experiments considered in this paper (see section Application to real experiments), we set *γ* = 2 pixels, which was the value given in [10] based on manual measurements of 150 endosomes. Then, λ was calculated for the sequence of scales {*R*_1_ = 1, *R*_2_ = 1.05, *R*_3_ = 1.10, …} stopping after three consecutive decreasing λ values.

## Algorithm evaluation with simulated endosomes

**Fig 2:**
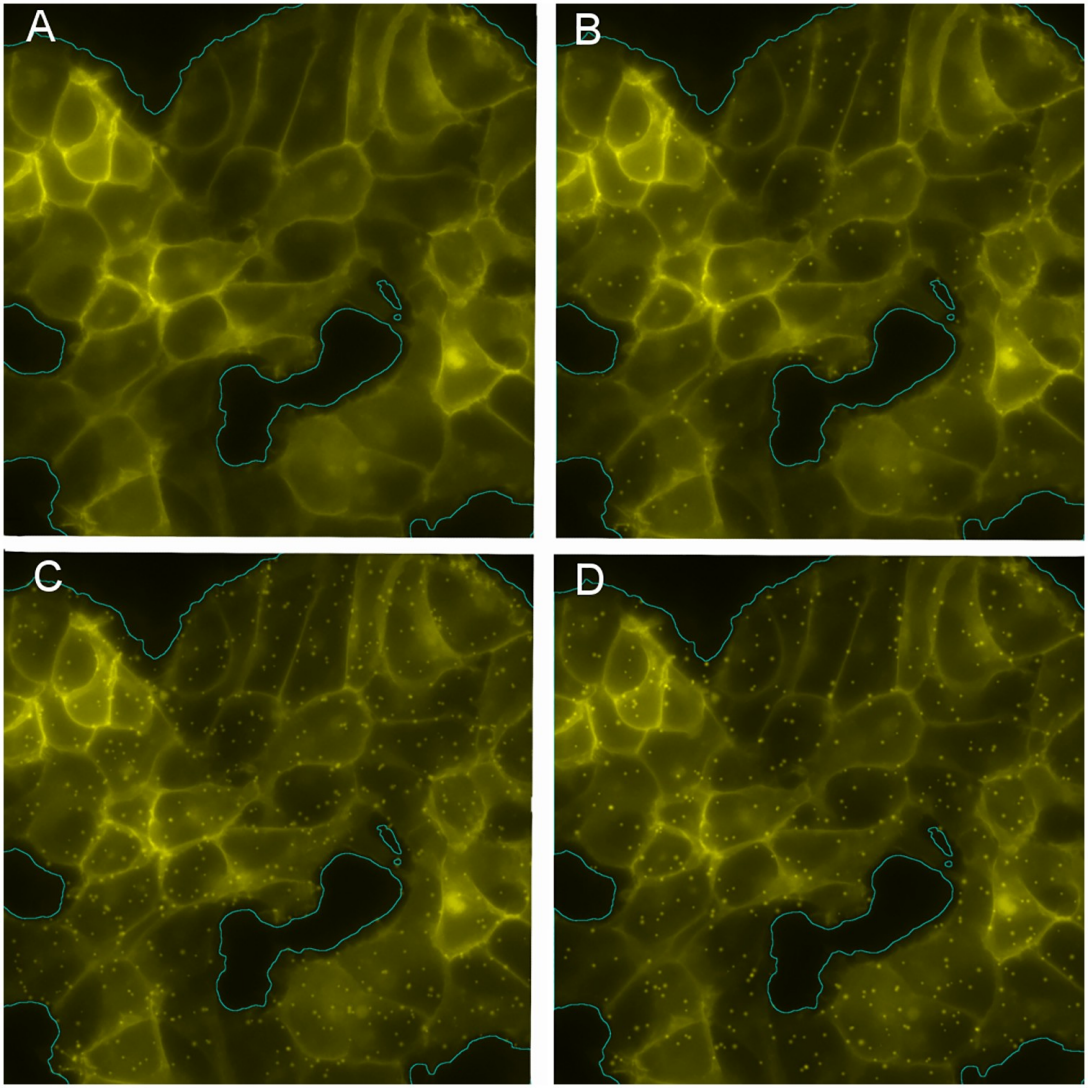
Simulated images. (A): Background image taken from the same experiment as in Fig 1 at *t* = 0. (B,C,D): Background image (A) plus simulated endosomes with *n* = 5, *A* = 2*s*, (B); *n* = 15, *A* = 2*s*, (C); and *n* = 10, *A* = 3*s*, (D).

Here we test the performance of our algorithm on simulated endosomes added to 17 background images, {*I*_0_}, (see Fig 2 and section Application to real experiments for details). The endosomes are added based on the following approximation:

Let *N* be the number of endosomes in image *I*_*t*_. The time-lapse images {*I*_*t*_} can be approximated as the sum of a constant background equal to *I*_0_ plus the sum of the contribution of the *N* endosomes. Note that no endosomes are present at *t* = 0 since the drug has not been added yet.

$$\begin{array}{cc} & I_{t}\approx I_{0}+E_{t}, \end{array}$$ (7)

$$E_{t}=\sum_{i=1}^{N}F_{i},$$ (8)

where the image *F*_*i*_, corresponding to the *i*-th endosome centered at a arbitrary position inside *B*_*t*_, is obtained by evaluating Eq 1 for all the pixels in *B*_*t*_.

We considered *n* = {5, 10, 15, 20} endosomes per cell and the number of cells, *n*_*c*_ in each of the 17 regions of interest {*B*_0_} was manually counted. Hence, *N* = *n* ⋅ *n*_*c*_ endosomes were simulated at random in each *B*_0_.

We assumed a uniform random spatial distribution of the endosomes in each *B*_0_, which is not realistic since in real images they appear to be clustered in the latter acquisition times. However, this should be a reasonable assumption for testing our algorithm since Δ*m* is a global variable that does not take into account the spatial distribution of the endosomes.

The endosomes were modeled using Eq (1) with *γ* ∼ *N*(2, 0.5) as estimated in the Supplemental Data of [10]. Three amplitude values were considered, namely *A* = *k* ⋅ *s* with *k* = {1, 2, 3}, where *s* is the standard deviation of *I*_0_ in the region of interest *B*_0_. Combining the four considered values of *n* and the three of *A*, we obtained a total of 12 sets of 17 simulated images *I*_*n*,*A*_ each. Since the total fluorescence of the image should be constant, we normalized each simulated image:

$$\begin{array}{ccc} I_{n,A} & = & \sqrt{1-s_{E}^{2}}\times \frac{I_{0}-\bar{I_{0}}}{s^{2}}+E_{n,A}-\bar{E_{n,A}}, \end{array}$$ (9)

$$\begin{array}{ccc} E_{n,A} & = & \sum_{i=1}^{n\cdot n_{c}}F_{i}\left(A\right), \end{array}$$ (10)

where $\bar{\left(\cdot \right)}$ is the sample mean in region *B*_0_ and $s_{E}^{2}$ sample variance of *E*_*n*,*A*_ in *B*_0_.

**Fig 3:**
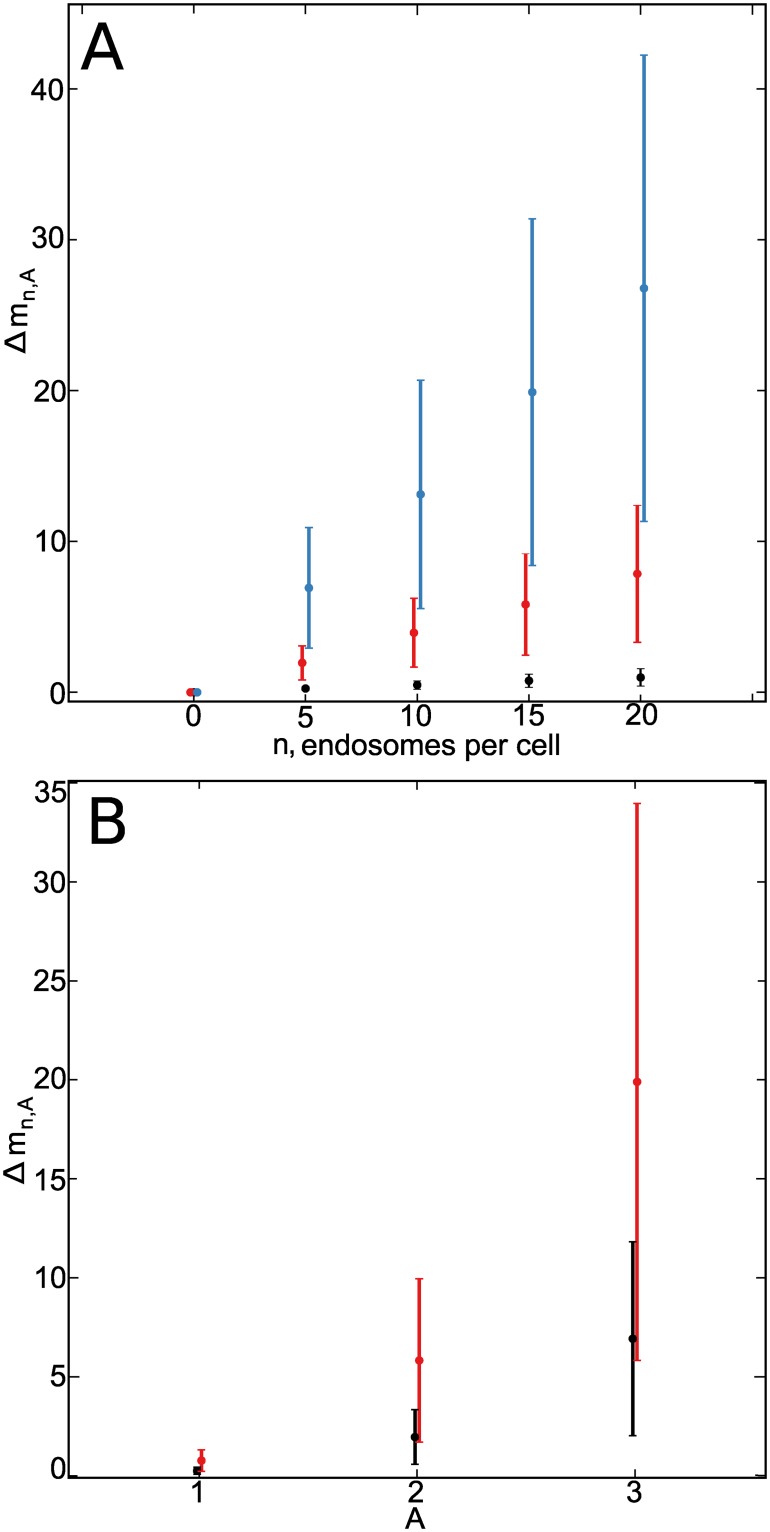
Results from simulated images. (A): Δ*m* variation with respect to the number of endosomes per cell, *n* for three fixed amplitude values, *A* = *s* (black), *A* = 2*s* (red) and *A* = 3*s* (blue). (B): Δ*m* variation with respect to *A*, for two fixed *n* values *n* = 5 (black) and *n* = 15 (red). 17 different background images were considered in each set of values {*n*, *A*}.

The algorithm was applied to simulated images *I*_*n*,*A*_ in an analogous way as we describe in Eq 4 for real images *I*_*t*_. Each image *I*_*n*,*A*_ was convolved with the LoG filter at optimal scale *R*_*opt*_, obtaining the convolved image $I_{n,A}^{\prime }$. Δ*m*_*n*,*A*_ was then computed for all the simulated images:

$$\Delta m_{n,A}=\frac{m_{n,A}-m_{0,0}}{m_{0,0}},$$ (11)

$$m_{n,A}=\frac{1}{n_{pix}}\sum_{\vec{x}\in B_{t}}(I_{n,A}^{\prime }\left(\vec{x}\right)-{\bar{\omega }}_{n,A}{)}^{3},$$ (12)

$${\bar{\omega }}_{n,A}=\frac{1}{n_{pix}}\sum_{\vec{x}\in B_{t}}I_{n,A}^{\prime }\left(\vec{x}\right),$$ (13)

where *m*_0,0_ = *m*_0_ is the value for the background image with no simulated endosomes. The results are represented in Fig 3.

Given the same number of endosomes per cell (Fig 3A), the values of Δ*m* were found to be significantly different for the three amplitude values *A* = 1, *A* = 2 and *A* = 3 (t-test, *p* < 0.05). In the case of simulated endosomes with the same amplitudes but different *n* (Fig 3B), we found that Δ*m* was significantly different comparing *n* = 5 with *n* = 10 and *n* = 10 with *n* = 20 (t-test *p* < 0.05). However, Δ*m* is not significantly different considering *n* = 15 versus *n* = 20 and *n* = 10 versus *n* = 15 endosomes per cell (t-test *p* > 0.05).

The simulated images with *n* = 10, and *A* = 2*s*, 3*s* were also used to compare the discrimination power of third and fourth order moment. Δ*m* was significantly different for both of them, but the p-value using the third order moment was significantly lower compared to the one obtained with the fourth order moment (6 ⋅ 10^−6^ and 2 ⋅ 10^−4^ respectively). The robustness of Δ*m* versus variations in the scale parameter *R* was also checked for this case. The p-value remained almost constant (3 ⋅ 10^−6^ < p-value < 9 ⋅ 10^−6^) for 1 < *R* < 2.5 pixels confirming the robustness versus small variations in *γ* or *R*_*opt*_.

## Application to real experiments

The experiments performed in [10] are used as a proof of concept to test the algorithm. Drugs diluted in physiological saline solution were perfused into the microscope chamber for internalization experiments in real time. Then, images were acquired in an inverted epifluorescence microscope. The initial image stacks consisted of *n*_*z*_ = 9 planes of 0.49*μ*m *z*-step size and *n*_*t*_ = 15 at a rate of 1 frame per minute. The 16-bit resulting images had a resolution of 1004 × 1002 pixels (0.13*μ*m pixel size). The maximum intensity *z*-projection was performed by selecting for each pixel *i* the maximum value across the *n*_*z*_ *z*-planes. Thus, the stack is reduced to a set of *n*_*t*_ time-course images $\left\{I_{t}\}=\{I_{0},I_{1},\ldots ,I_{n_{t}-1}\right\}$. Materials, receptor fusions with fluorescent proteins, generation of stable Flp-In T-REx HEK293 cell lines, cell transfection and living cell epifluorescence microscopy are detailed in [10].

The Δ*m* algorithm was applied to two sets of experiments, which we call -DOX and +DOX as in [10]. Mu opioid (MOP) receptor was tagged at the carboxy-terminus with yellow fluorescent protein (YFP) and permanently expressed in Flp-In T-Rex HEK293 cells. The +DOX experiments were conducted in cells pre-treated with doxycycline (0.01*μg*/*ml*) for 24 hours prior to microscope observation, in order to induce the expression of c-myc-5 − *HT*_2*C*_-Cerulean receptors together with MOP receptors. In total 19 experiments were considered in the -DOX condition, they consisted of four different treatments, namely morphine (10*μ* M), methadone (10*μ*M), sufentanyl (1*μ*M) and DAMGO (H-Tyr-D-Ala-Gly-N-MePhe-Gly-OH, 10*μ* M). The +DOX set consisted of 20 independent experiments with one additional drug combination, morphine (10*μ* M) + serotonin (5HT, 10*μ* M).

Δ*m*_*t*_ was calculated with Eq 4. Two possible time responses of Δ*m*_*t*_ were expected, namely a sigmoid response in drugs inducing endocytosis and a flat, linear response for drugs unable to induce endocytosis such as for instance morphine. Therefore two regressions were performed on each Δ*m*_*t*_, namely a linear fit and a sigmoid function fit. The following sigmoid function was used:

$$\begin{array}{c} \mu_{t}=\mu_{0}+\frac{E_{max}}{1+\text{exp}[\alpha \cdot (t_{1/2}-t)]}, \end{array}$$ (14)

where *E*_*max*_ is the maximum response or efficacy, *α* the slope, and *t*_1/2_ the time needed to reach 50% of the maximum response.

**Fig 4:**
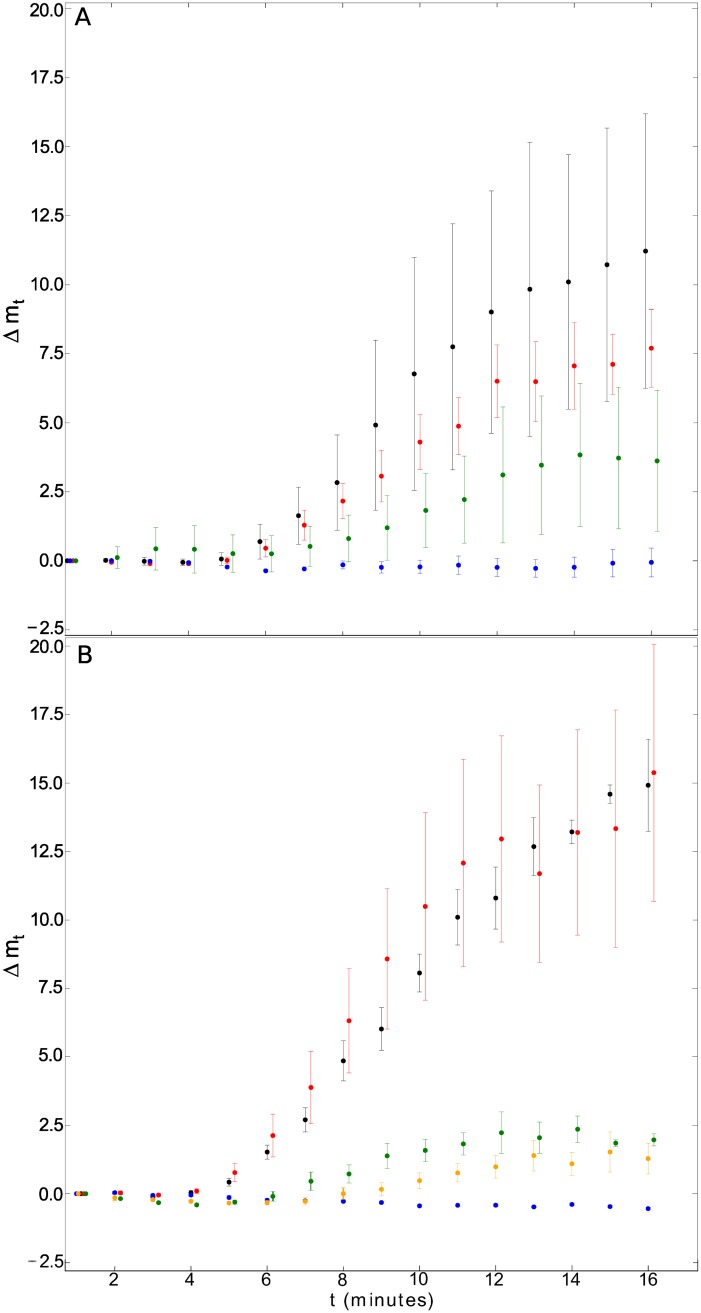
Results from real experiments. (A): Evolution of Δ*m* through time after treatment with DAMGO (black), sufentanyl (red), morphine (blue) and methadone (green) (-DOX Condition). The number of experiments per drug was 5, 5, 3 and 4 respectively. Each point represents the mean ± SEM. (B): Corresponding results from +DOX Condition, including morphine+5HT (orange). The number of experiments per drug was 5, 4, 3, 4 and 2 respectively.

The goodness of fit was evaluated using the standard coefficient of determination, $r_{lin}^{2}$, in the linear case, and coefficient of determination, $r_{sig}^{2}$, in the sigmoid function fit:

$$r_{sig}^{2}=1-SS_{res}/SS_{total},$$ (15)

$$SS_{res}=\sum {\left(\Delta m_{t}-\mu_{t}\right)}^{2},$$ (16)

$$SS_{total}=\sum (\Delta m_{t}-\bar{\Delta m}{)}^{2},$$ (17)

where the summations run from *t* = 0 to *t* = 15 minutes. In order to exclude experiments with important systematic artifacts, only experiments with $r_{lin}^{2}>0.5$ or $r_{sig}^{2}>0.5$ were considered. Two experiments in each set, -DOX and +DOX, were discarded. The presence of systematic artifacts was confirmed by visual inspection. These artifacts were usually observed in the initial time frames as irregular brightness fluctuations which were clearly not related to the biological response. For the rest of experiments the obtained results can be seen in Fig 4. The results show that Δ*m*_*t*_ has a sigmoid dependence with time, with the exception of the morphine experiments, where a flat response is observed, as expected from the results in [10].

A multivariate ANOVA (MANOVA) was carried out with R. The vector of variables (*E*_*max*_, *t*_1/2_, *α*) was considered for the experiments with $r_{sig}^{2}>0.5$. The type of drug (DAMGO, sufentanyl, morphine+5HT or methadone) and treatment with doxycycline (-DOX or +DOX) were considered as non-random factors. The analysis revealed a significant difference only for the drug factor (*p*-value <0.01). No significant differences were detected for factor doxycycline (*p*-value > 0.1). Separate ANOVA analyses indicated that the method difference was due to *E*_*max*_ and *α* (*p*-value < 0.01), whereas no significant differences were found in *t*_1/2_ (*p*-value > 0.1).

The mean ± Standard Error of the Mean (SEM) values of variables (*E*_*max*_, *t*_1/2_, *α*) are summarized in Table 1. Morphine experiments are not listed since they failed to fit a sigmoid curve. The values in the table were calculated for -DOX and +DOX experiments together, since the DOX factor did not present significant differences.

**Table 1:** Sigmoid curve fitting parameters.

	*E*_*max*_	*t*_1/2_, min	*α*
DAMGO (10)	16.1 ± 3.1	10.4 ± 0.6	0.53 ± 0.06
Sufentanyl (9)	11.1 ± 2.5	9.1 ± 0.3	0.62 ± 0.05
Methadone (6)	3.3 ± 1.3	9.7 ± 0.7	0.84 ± 0.11
Morphine+5HT (4)	1.8 ± 0.7	9.7 ± 1.0	1.32 ± 0.37

Obtained for the data plotted in Fig 4. Mean values ± SEM of (n) independent experiments are listed. Only experiments with $r_{sig}^{2}>0.5$ were considered.

## Discussion

In this report we propose a new algorithm to quantify pharmacological responses in fluorescence microscopy images by calculating the third order moment increment over time after convolution with a Laplacian of Gaussian filter at optimal scale. Receptor endocytosis stimulated by agonist drugs [10] has been used as a proof of concept to validate this methodology.

Data obtained with the algorithm from simulated images resulted in a significant statistical difference, and show that it is possible to discriminate on both, number of endosomes per cell and endosome fluorescence intensity. It has been usually observed in real data that an increase in the number of endosomes is accompanied by an increase of endosome fluorescence intensity across the experiment.

Significant differences in the pharmacological response of drugs used as agonist compounds were observed after applying the algorithm to real data. Morphine was unable to promote MOP receptor endocytosis and its response fits to a flat line, whereas DAMGO, sufentanyl, methadone and the combination of morphine plus 5-HT showed a sigmoid time response curve.

A vector of parameters (*E*_*max*_, *t*_1/2_, *α*) was obtained for each experiment through a sigmoid curve fit. A multivariate ANOVA detected a statistically significant difference in the parameter vector attending to the drug factor, whereas no significant differences were found for the factor doxycycline. This was to be expected since the treatment with doxycycline only activates the inducible expression of c-myc-5 − *HT*_2*C*_-Cerulean receptors and should not affect MOP receptor endocytosis from a pharmacological point of view. Individual one-way ANOVA subsequent tests indicated that the difference among drugs was due to *E*_*max*_ and *α* parameters.

The proposed method does not rely on the manual annotation of images nor on a manual characterization, both of which are slow, tedious and could introduce bias. Moreover, it improves the Q-endosomes algorithm [10] since the results do not depend strongly on parameters that have to be set manually and it provides information attending to the intensity of the endosomes, not only on their number.

The above qualities make this algorithm suitable for drug screening with exploratory purposes in automatic microscopy, but the same principle can be easily extended to similar problems where the high number of experiments requires fast and non supervised methods and segmentation algorithms do not carry a complete solution for detecting the objects of interest. Our method could also be applied to multi-spectral fluorescence images, by adapting the algorithm for multiple channels.

We have centered on validating the algorithm for kinetic experiments, the dose-dependent response will be analysed in future work. Future studies could be focused on analysing the variation of size and spatial distribution of endosomes depending on the evaluated drug. In the present work the spatial distribution is irrelevant and the endosome size is assumed to be independent regarding the agonist drug used.
